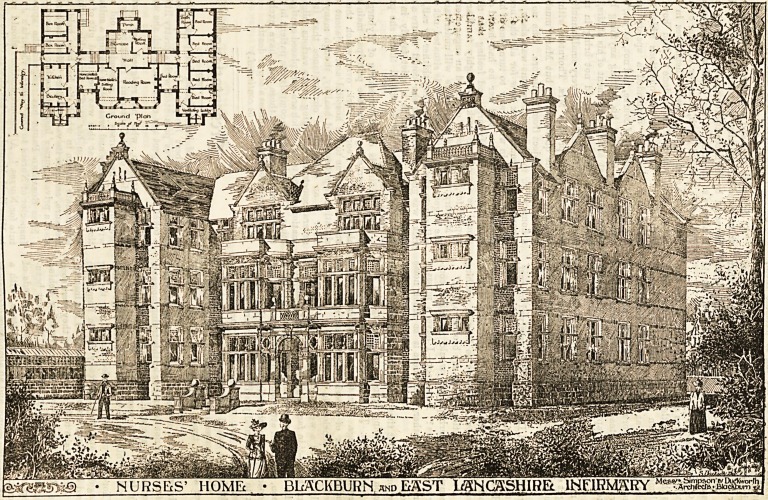# The Hospital Nursing Supplement

**Published:** 1891-10-31

**Authors:** 


### "The Hospital" Nursing Mirror.

Being the Extra Nursing Supplement of "The Hospital" Newspaper.

Contributions for this Supplement should be addressed to the Editor, The Hospital, 140, Strand, London, W.C., and should have the word "Nursing" plainly written in left-hand top corner of the envelope.

### En Passant.

A BOURNEMOUTH BAZAAR.—In aid of the Bournemouth Nurses' Institute a bazaar was recently opened by the Countess of Malmesbury. The Mayoress held one stall, and another stall was devoted to articles for sale by Nurses of the Institute, five of whom were in attendance. A number of prettily dressed dolls, one in particular attired as a bride, and some to represent nurses (with the distinctive badges "B.N.I."), fancy shawls, sofa cushions, work baskets, a gipsy table made by Miss Hincks (Lady Superintendent of the Institute), and a number of woollen articles. The proceedings were very successful.

SHORT ITEMS.—We learn with regret that Miss Barton is leaving the Royal Free; she is one of the most courteous and popular of hospital Matrons. This is a good post vacant, and there will be keen competition for it.—All letters with regard to the Pension Fund should be addressed the Manager, Royal National Pension Fund for Nurses, 8, King Street, Cheapside, and not to any official by name.—The editorial sanctum is lined with drawings of skeletons; the award will be announced and the prize sketch reproduced in our issue for November 7th.—The second edition of Dr. Lewis's "Text Book of Nursing" is just out, and orders can now be attended to.—The Windsor Coroner on Saturday committed Dorothy Davis, a midwife, for trial, upon the charge of wilfully murdering Annie Simpson, a domestic servant, upon whom she had performed an illegal operation.—Two monthly nurses willing to attend middle-class cases send us their addresses, but we cannot at present undertake to organise this movement.—Worcester Infirmary has affiliated with the Royal National Pension Fund for Nurses.

GOVAN GOES FORWARD.—The Parochial Board of Govan lately invited those interested to inspect and assist in opening the new nursing arrangements at the Merryflats Hospital. Ex-Bailie Hamilton Mair presided, and said it was over 20 years since they occupied the premises they had been examining, and for that period the system of nursing was that known as pauper nursing, presided over by a Matron and under-Matron. Of recent years a very great improvement had taken place in nursing, and two years ago, when Mr. Mair was Chairman, he drew attention to the matter, with the result that a committee was appointed and visited the various institutions of a similar kind in Glasgow, Edinburgh, Paisley, and Greenock. It was agreed to adopt the employment of trained nurses, believing that the inmates received greater attention and comfort, that nourishment and stimulants were more regularly and carefully administered, that cases of imposture were more readily detected, and that temporary and effective aid were rendered in cases of sudden emergency. At a cost of nearly £500 the hospital had been rearranged, and it was the desire of the Board that the nurses might find in their apartments every comfort that home could give. They had been fortunate in securing the services of Miss M'Donald as Lady Superintendent, who had entered upon her duties. Miss M'Donald had been successful in securing a staff of most efficient nurses. The average sick in the institution numbered 180 to 200, and they had a nursing staff of eight. The nurses quarters are nicely fitted, and a piano is provided.

ENTERTAINMENTS FOR NURSES.—Guy's Hospital has set a good example by starting a series of entertainments for nurses, at the first of which our old friend, Mr. Corney Grain, gave his delightful sketch, "At the Pantomime." There was a large gathering of nurses present in the Court Room, including some from the Evelina and the Waterloo Road Hospitals, and they seemed to thoroughly enjoy themselves. There was plenty of good music, and the students who organised the entertainment are to be congratulated and applauded. It is proposed to give a concert every month.

SAINT HELENS.—In the middle of the grimy land of collieries, chemical works, and Beecham's pills, stands St. Helens, which has lately roused admiration by its unique hospital system. There is the usual Municipal Hospital for infectious diseases; there is the Providence Hospital, worked by Sisters of Charity, who go round begging for subscriptions; and there is the Cottage Hospital, supported by the pennies of the working men. It is the contrast of the last two which is so wonderful; the first redolent with the spirit of the middle ages and ecclesiasticism, and the second redolent of Trades-Unionism and the nineteenth century. Standing in the same town they afford a strange subject for speculation, for they are both doing excellent work. We wonder why both are boycotted by the "Hospital Annual"?

THE NAME OF "NURSE."—"I have here," said the Professor, "a rare object of the past which we will proceed to examine; it is a well preserved specimen of the nurse of the end of the nineteenth century. You will observe that the head is fairly well developed, the brain of the ordinary weight of that period; the eyes are small, the mouth is big. The heart is, indeed, a hollow muscle, small and shrivelled; the lungs are normal. But the stomach is enormous; this huge bag which I hold before you seems to have filled almost the whole internal economy of a nurse, to the deterioration of all other organs. Observe, also, the size of the æsophagus and the intestines, and you will understand that this curious creature must have obtained her name of nurse, nourrice, from her love of nutriment or food."

BLACKBURN INFIRMARY.—For the office of Matron to this institution, which accommodates about one hundred beds, there are over fifty applications to fill the place vacant, owing to the death of the late Miss C. Prince, as noticed in a recent issue of The Hospital. The House Committee met on Wednesday at the Infirmary, and went through the applications, and reduced them to a limited number. It is expected that the appointment will be made on Monday, November 2nd, at the ordinary monthly meeting of the Board. The Infirmary is well situated, and occupies a pleasant position in the centre of large and well laid-out grounds, and is well managed by a Board interested in the work, and who are well supported by the whole district. The position of the Matron is a desirable one, and will be improved when the proposed Nurses' Home is completed, as some relief in the duties will in all probability be arranged. When Miss Prince died £20 were due to her as salary. Her sisters, who are the executors under her will, knowing the deep interest she felt in the charity, have determined to present the amount due to the Nurses' Home, in which some permanent memorial of the late Matron will be placed.

### Glasgow Infirmary Nurses.

It seems that the disturbance at Glasgow is not ended by the publication of the official report (which we deal with elsewhere); for since its appearance the resident medical officers of the Infirmary, in a spirit of chivalry, have issued a protest against the conclusions arrived at by the Committee. Ten of these young gentlemen sign their names to the protest and undertake to prove certain facts with regard to the nurses' hours which, if known to the management, must be considered in the highest degree discreditable. We have not the slightest sympathy with those nurses who complain of cold dinners on Sunday—we see no reason why the nurses at Glasgow should not have hot dinners on that day as other nurses do, but the subject is not worthy of the tremendous fuss being made about it; but the question of overwork is quite another matter. Here are some of the facts for the accuracy of which the resident medical staff are ready to vouch:—

| Hours on Duty. | Hours | | Hours for Rest and Recreation. |
|---|---|---|---|
| Nurse A. | | | |
| 7.30 a.m. to 6.30 p.m., 9 p.m. to 11 a.m. | 27½ | less | 2½ |
| Nurse B. | | | |
| Aug. 17.—7.30 a.m. to 8.30 p.m., 11.30 p.m. to 4.15 a.m., 11.30 a.m. to 8.30 p.m. ............ | 37 | less 3 + 7¼ = | 10¼ |
| „ 20.—7.30 a.m. to 8.30 p.m., 1.30 a.m. to 7 a.m. 2 p.m. to 8.30 p.m. ........................ | 37 | „ 5 + 7 = | 12 |
| Sept. 1.—7.30 a.m. to 8.30 p.m., 12.30 a.m. to 8 a.m. .................. | 24½ | less | 4 |
| Nurse C. | | | |
| Aug. 2.—7.30 a.m. to 5 p.m. 9.45 p.m. to 10 a.m. ........................ | 26½ | | 4¾ |
| „ 25.—9.45 p.m. to 1.15 p.m. next day (less ½ hour) ............ | 15½ | „ | ½ |
| Sept. 1.—7.30 a.m. to 4.15 p.m., 9.15 p.m. to 11 a.m. (less ½ hour) | 27½ | „ | 5 |
| And for several days just after 1st September this nurse was on duty daily, 7 30 a.m. to 10 p.m. | | | |

If it is known that a special nurse will be required during the night for a bad case, the common practice is to send a probationer (who has been up since 6 a.m. and on ward duty since 7.30 a.m.) to bed about 5 p.m., call her up at 9 p.m., and she remains on duty till 10 or 11 a.m. the next day. The nurse is rarely able to rest between 5 p.m. and 9 p.m., owing to the noise of Castle Street traffic.

Medical Advice Concerning Nurses' Health.—This is sometimes disregarded if the officials can conveniently do so, and the doctor's back is turned.

Deduction of Nurses' Salary while on Sick Leave.—This is dependent on the pleasure of the officials. Judging from known cases, there appears to be no definite plan followed in making or not making the deduction.

The nurses have to make use of the lavatory and closet accommodation provided for the patients, no matter what diseases the male or female patients may be suffering from. If one of the side-rooms in each ward were given for the nurses' use, this most objectionable arrangement could be easily remedied with little outlay of money.

These are legitimate causes of complaint, and the managers in their report have promised the desired amendments. We cannot see the injustice of fining nurses for broken thermometers, for it is a fact that in one hospital thermometers to the cost of £1 a-week used to be broken till every break had to be explained to the Matron, who made a small fine in cases of carelessness; now the cost to the hospital of thermometers is only about 4s. a-week. Obviously we have yet to hear the conclusion of the whole matter. The Committee cannot sit silent under the serious and painful accusation that they have persistently worked some of their nurses 24 hours out of 36.

Royal Red Cross.—The Queen has conferred this decoration on Nora Henrietta, Lady Roberts, Mrs. Caroline Rebecca Damant, Mrs. May Emma Cawley, Miss Catherine Grace Loch, Miss Edithe Welchman, Miss Elizabeth Mary Lickfold.

### Blackburn Nurses' Home.

We publish this week an illustration of the Nurses' Home to be built in connection with Blackburn Infirmary. Since every hospital must now recognise the right of nurses to be properly housed, and many committees are only waiting for funds to commence building Nurses' Homes, we believe that a description of this useful and pretty building will be of a wide interest. Before drawing their plans the architects took the trouble to visit other institutions, particularly the Home in connection with the London Hospital, and have made all arrangements according to practical needs.

The building will be a structure of red brick, three storeys high, of a style suited to that of the Infirmary. The ground plan shows a building consisting of three parallel blocks, connected by a corridor which runs through the entire structure. The middle block is much shorter than the two end ones. The covered way will run from the central block of the Infirmary. This covered way will, of course. be almost entirely used by the nurses going to and from the Infirmary. The main entrance to the home will be by a porch in the central block, and there will be a waiting-room on the left of the entrance hall. Across the corridor, and at the other end of the block, will be the reading-room, 20 ft. by 15 ft. On the right in the same block, will be the home sisters' sitting room, and next to it will be the home sisters' bedroom, both of which the nurses will have to pass to get to their own rooms. This should assist in maintaining good order. In the wing on the right will be the kitchen, scullery, box rooms, and the usual conveniences. The wing on the left will be entirely taken up with bedrooms and a bathroom for six private nurses. Each bedroom, both on this floor and on the other floors, will contain dressing table with drawers, a couple of chairs, a single bed, and a wardrobe, and will be supplied wth hot and cold water. Picture moulds will be fitted, and the walls will be finished in delicate tints with Morse's calcarium. The room floors will be covered with 1½ inch pitch pine wood blocks, and in the sitting-rooms and bedrooms they will be finished with a scraper and polished with bees' wax and turpentine. The entrance hall and vestibule will be cased to a height of four feet with panelled framing, and all the internal woodwork will be pine, stained and varnished. The floors of the corridors and staircase landings will be finished in granite concrete, with coloured margin, rubbed and polished. The corridor walls will be lined with red or brown enamelled bricks, finished to the ceiling with white, buff, or cream-faced bricks. On the first floor there will be six bedrooms and a bathroom in the right wing for probationers, and in the left wing the same number of bedrooms and a bathroom for charge nurses. The central block will be taken up with a recreation-room, 20 ft. by 15 ft., from which access to the balcony will be gained by two French windows. Next to it there will be a spare room, and on the other side of the corridor a larger linen room. On the second floor there are to be bedrooms for six probationers in the right wing, and for six night nurses in the left wing. In the centre will be placed the servants' bedrooms, another spare room, and a clothes-room. The heating of the corridors, waiting-room, and linen and clothes-room will be by hot water from a boiler in the basement, and the other rooms will be warmed by ordinary fires. Fanlights for ventilation will be fixed in the corridors over all doors. The architects' estimate of the cost is made up as follows:—139,950 cubit feet of building at 5½d., £3,207; cellar, £80; foundations, £90; covered way, £100; total, £3,477.

### Appointment.

Ingham Infirmary.—Miss Ethel Rimington has been appointed Matron to the Ingham Infirmary and South Shields and Westoe Dispensary. She trained at Leeds, and for the last year has acted as nurse at the Dreadnought Hospital, Greenwich.

• NURSES' HOME • BLACKBURN AND EAST LANCASHIRE INFIRMARY Messrs. Simpson & Duckworth, Architects, Blackburn.

### A Nurse in Natal.

When Mary Goodricke and her aunt reached the end of their voyage they were much struck by the beauty of the bay in which they found themselves, and where they had to wait for the tug to come and land them. Mary felt confident her brother would come out on the tug to welcome them, and meantime she was very content to wait. Poor Mary! these were the last moments of unmixed happiness she was perhaps ever to know. What a merciful provision is that which hides the future from our ken! If we knew what lies before us of sorrow, disappointment, or failure, should we ever find courage to go upon our way?

Towards noon, the tug was seen steaming out of the harbour towards the Garth Castle, and, on reaching the vessel, several gentlemen came on board. One of them (whom Miss Goodricke could not at that time have recognised as a medical man, for he wore a loose light suit, and broad brimmed straw hat instead of the conventional black) approached her with the captain, who introduced him as Dr. Jones.

"You are my brother's friend?" said she, interrogatively, with the bright smile which was one of the characteristics of her otherwise rather plain face. "Was Robert not able to come out?"

"No, he was not," said Dr. Jones, in a reserved manner, which Mary thought rather unnecessary under the circumstances. "He could not – in fact—I have come myself to see you landed, and put safely in the train."

"That is very kind of you. Shall we be long in reaching the Berea? I am so impatient to see my brother again."

"Yes, of course; but perhaps you would not mind coming below for a few minutes? I—in fact—I have a message to give you from your brother, and there is so much noise and confusion here."

It was now for the first time that Miss Goodricke felt an uneasy suspicion of something wrong; and leading the way to the saloon, where Mrs. Thornton already was, she soon heard the sorrowful tidings. Dr. Goodricke had been taken ill, with a serious attack of enteric fever, that scourge of unacclimatised young bachelors in Natal.

He had only been ill a week, but the attack was of so severe a nature that—"in fact, we fear the worst," said Dr. Jones abruptly. The doctor was at heart a truly kind and benevolent man, and much attached to his young partner, but his nervous consciousness of the pain he was inflicting, caused him to speak in an unnaturally stiff and awkward manner.

"Of course, I knew you were expected," he went on. "Poor Goodricke has talked of little else for the last three months, and when the vessel was signalled, I determined to leave him for a short time, while I came out to tell you myself."

Miss Goodricke's habits of control, and the nurse's instinct of self-forgetfulness did not desert her at this trying moment. If her brother were ill, she was now at hand to nurse him, and while life lasted there was hope! Was not this the nurse's creed? So, while the slow quiet tears of old age rolled down Mrs. Thornton's cheeks, she busied herself in gathering everything together, and making ready for an immediate start. Arrived in Durban, where Dr. Jones had left instructions for his own carriage to be in readiness, they were soon driven to the Berea, a natural plateau, stretching behind and above the town, where, embowered in the most beautiful semi-tropical luxuriance of growth, lies thousands of suburban homes. Here, in a pretty little cottage, with a broad verandah running round, and an abundance of creepers and gorgeously blooming flowers, Mary found her brother, already far past recognition of her. It was soon evident to all that the closing scene was at hand. Not all the secrets of the doctor's science, or of the nurse's art could arrest the life that was passing away, and before the sun had set upon her first afternoon in Natal, Mary had lost the last remaining member of her own immediate family. There was an end of all their plans for the future!

Their brief dream of happiness was over, and they were rudely awakened to find themselves without a home in a foreign land, and deprived of that strong support around which all their hopes and affections had centred.

At first both aunt and niece were overwhelmed by such an unlooked-for calamity, the terrible nature of which seemed increased by the necessity for almost immediate burial which is always so shocking to new residents in tropical climates. The active spirit of youth, however, and her own self-dependent nature soon began to assert themselves in Miss Goodricke. What was now to be done? And how were they both to live? These were the questions which forced themselves upon her, and demanded a speedy answer. Dr. Goodricke's practice, of course, had not, as yet, been of such a nature as to allow of his putting anything by; and his sister found that after a settlement of his affairs, the sale of his furniture, books, &c., would not bring in more than a meagre sum. This, added to a very small annuity which she possessed, would provide Mrs. Thornton with board and lodgings in some quiet place; but Mary must work, and with a different object from heretofore. She no longer had a home to go to when employment was slack, or rest was necessary; she must work now for all the needs of life. While hesitating in which way to turn, one of her brother's medical acquaintances told her he had heard of a vacancy in the hospital at Myburg, and feeling that this, at least, would be a mode of work thoroughly familiar to her, she applied for, and speedily obtained an engagement there.

Myburg, as some of my readers will understand, is one of the largest towns in Natal, and is situated not one hundred miles from the Port. Travelling thither by rail, Miss Goodricke was glad to find that she could secure a comfortable room for Mrs. Thornton in a quiet family in the town; and having seen her a little settled there, she proceeded to White's Hospital.

This building is on the outskirts of Myburg, occupying some leven bordering upon the principal main road. It is also close to the People's Park, over which the fresh coast breezes are borne to the hospital and adjacent town. The situation is pleasant and healthy, though many would consider it an unfortunate circumstance that it adjoins the several cemeteries belonging to the various religious denominations of the town, which are here placed contiguous to each other. This arrangement compels the patient in the hospital to overlook the graves of those who have exchanged their beds of pain for the dreamless sleep of death.

*(To be continued.)*

### Presentations.

On the occasion of her resigning her post as Senior Sister at the Royal Albert Hospital, Devonport, Miss E. C. Fry (Sister Norman) was presented with a pretty little clock, as a token of the respect and esteem in which she was generally held.

Miss Harbord, who has resigned her post as Lady Superintendent of the Canterbury Institute, was, on leaving, presented by her nurses with a handsome old oak bureau, with brass handles and brass plate bearing a suitable inscription. The fact that this was the united gift of every nurse and probationer on her staff is a valuable testimony to the affectionate esteem in which she has been held, and will, we feel sure, enhance its value to her. Miss Harbord is going to start a private nurses' institute on her own account.

Miss Rosalind Paget has resigned the appointment of Inspector of Nurses to the "Queen Victoria's Jubilee Institute for Nurses." All communications should now be made to Miss E. M. Mansel, who has charge of the work of inspection. The Queen has been pleased to appoint Miss Rosalind Paget to be a member of the Council of the Institute, and has approved of the recommendation of the Council that the collar and pendant should be presented to her in recognition of her valuable services to the Institute.

### Everybody's Opinion.

*[Correspondence on all subjects is invited, but we cannot in any way be responsible for the opinions expressed by our correspondents. No communications can be entertained if the name and address of the correspondent is not given, or unless one side of the paper only be written on.]*

### NURSES' FOOD.

MISS EMMA DURHAM writes: Much has been written and said about nurses and their food, but nothing beyond giving the diet of various hospitals has been done to remedy the food question. All sailors and soldiers in Great Britain and Ireland, I believe, have the same diet, that is to say, the diet varies little in the different barracks, and in all large establishments there must be a uniform diet. I am not acquainted with the usual sums which hospitals generally allow for feeding the nursing staff, but I would suggest that a committee of clever and experienced men and women be formed to draw up a diet chart which shall be wholesome, nourishing, palatable, and economical, and that this diet be given in every hospital in the United Kingdom. Surely, no woman who offers herself for training expects luxuries out of money subscribed by people, many of whom go without luxuries themselves to be able to give to their poorer fellow creatures, and this fact of living out of part of voluntary subscriptions is, perhaps, not sufficiently considered by many who seek in nursing a means of livelihood which shall be interesting, remunerative, and socially approved. Nurses, and I mean all people engaged in the care of sick folk, be they Matrons, Sisters, nurses, or probationers should all have their meals together (in relays) with the exception, perhaps, of afternoon tea. If all hospitals were fed alike it would do much to lessen the prevailing discontent. Should there be a sufficient number of wealthy people who desire hospital training, one London hospital might be set apart for their reception and training—and in the matter of food they might contribute a certain sum per week, or quarter, as is done in a military mess, and have their food provided for them near the hospital, or provision might, perhaps, under the circumstances, be made for the accommodation of the necessary servants and culinary work within the hospital building.

### NURSES AS PATIENTS.

"E." writes: I can endorse all that "S." says about the brusque and unfeeling manner in which some who train for nurses perform their duties. I was a patient myself for a month in one of our London hospitals, and felt keenly the same things that "S." remarks upon. If we nurses could bear in mind the golden rule, "Do as you would be done by"—it is a very short rule—but what a grand difference the universal practice of it would make, and how comforting the thought would be to us, when on a sick bed, if, in looking back on our nursing lives, we could feel we had cheerfully and conscientiously done all we could for each sick one we had nursed.

### Notes and Queries.

### Queries.

(8) *Iron-mould.*—How can I remove stains of iron-mould from linen?—*Hospital Nurse.*

(9) *Chilblains.*—Can anyone tell me how to prevent or cure chilblains?—*Reader.*

(10) *Home Wanted.*—For a boy twelve months old; the father is mentally afflicted, and the mother cannot support the child.—*M. Campbell.*

(11) *Convalescents.*—Is there any association for helping convalescents to find work?—*J. E. L.*

### Answers.

(5) *Home for Epileptic.*—Apply to Miss Power, 29, Upper Montagu Street, W.

*"A Witness" and others.*—Next week; no room this.

*M. Taylor.*—Cullingworth's "Manual for Monthly Nurses," published by Churchill, price 1s. 6d.

*A Trained Nurse.*—We cannot print letters written on both sides of the paper.

*Sister Ellen R.*—Your query is an advertisement in disguise.

*Tuggs.*—No hospital will take you for two days a month. Understand that a woman who has not had a year's experience in a good hospital has no right to call herself a nurse.

*An Old Nurse.*—Rita, of Royal Infirmary, Truro, would like to have further particulars about your nurse's diet as given in a letter in THE HOSPITAL of October 17th.

*Christmas Competitions.*—Parcels received from Nurse Hitchcock, Annie Coates, P. Lockyer, and A Private Nurse.

*Certificated Nurse.*—Go to the Clapham Maternity Hospital for three months; fee, ten guineas. Write to the Matron, Maternity Hospital, Jeffrey's Road, Clapham, S.W.

*A. M. Smith.*—Get "Practical Electro-Therapeutics," published by Sampson Low, price 1s. 6d.

### A Heroine Discovered.

WE are indebted to the *Daily News* for bringing to light the story of Mdlle. Louise de Beaulieu, possessor of a military medal and eight medals for saving life. When the war broke out twenty years ago she joined a regiment as a vivandière. She was in eight battles, picked up under fire many soldiers, and was near being shot at St. Denis by the French as a spy. Her aristocratic air struck some soldiers, who took her prisoner and kept her in a hole in the ramparts till she could be tried. The trial was by a drum-head court martial, which sentenced her to be shot. On being taken to be executed she refused to let her eyes be bandaged, and asked as a last privilege to be allowed to give the word to fire. An officer admiring her pluck saved her. She was in the fights at Nanterre, Lebourget, Bry-sur-Marne, and Villiers, and always kept in the van. She attended at one of these places to twenty-five wounded men, and lost her right arm at Champigny while she was carrying a soldier to an ambulance. This did not disable her from work. Her record is one of the most splendid that man or woman could wish for. Though so badly wounded she was up and about and helpful at Groslay, Drancy, and in the sortie of Buzenval. One of her feats was saving a child from the sixth floor of a house which took fire in the Rue Saint-Honoré. She spent £800 in the terrible winter of the war in procuring comforts for the wounded whom she nursed in ambulances. Now, in the fiftieth year of her age she is earning her living by selling matches in the streets of Paris, having spent her small fortune in the cause of the sick and wounded.

### A Bed for a Sick Nurse.

BETWEEN October 20th and 27th we received the following sums towards endowing a bed at the Brassey Home, St. Leonards, where a poor seedy or convalescent nurse could go for rest and change, free of charge. We want some more friends of nurses to come forward as guinea subscribers: Sister Lund, £1 1s.; a Private Nurse, 16s., making one guinea when added to her previous subscription; and "A Friend," through a Private Nurse, £1 1s.; J. R. D., 1s.; E. M. J., 4s.; E. Marshall, 1s.; E. Bishop, 1s.; One of the First Thousand (Elmham), 1s; a Country Nurse, 1s.; Two Private Nurses, 1s.; and One of the First Thousand (Renshurst), 2s.; making fourteen guinea subscriptions, and £1 11s. 6d. in small sums, leaving fifteen guineas still needed.

### Amusements and Relaxation.

### SPECIAL NOTICE TO CORRESPONDENTS.

**Fourth Quarterly Word Competition commenced October 3rd, ends December 26th, 1891.**

Competitors can enter for *all* quarterly competitions, but no competitor can take more than *one first prize* or *two* prizes of any kind during the year.

Proper names, abbreviations, foreign words, words of less than four letters, and repetitions are barred; plurals, and past and present participles of verbs, are allowed. Nuttall's *Standard* dictionary only to be used.

The word for dissection for this, the FIFTH week of the quarter, being

"PHARMACY."

| Names. | Oct. 24th. | Totals. | Names. | Oct. 24th. | Totals. |
|---|---|---|---|---|---|
| Lightowlers | 66 | 112 | Jenny Wren | — | — |
| Bonne | 83 | 137 | Darlington | 78 | 126 |
| Morico | 86 | 141 | Nurse G. P. | 64 | 99 |
| Robes | 56 | 92 | Hetty | 53 | 58 |
| Dulcamara | 71 | 117 | Janet | 70 | 103 |
| Psyche | — | 7 | Jackanapes | 49 | 76 |
| Agamemnon | 81 | 134 | Ex Nurse | 51 | 51 |
| Nurse J. S. | 63 | 117 | | | |

### Notice to Correspondents.

*Jackanapes.*—Dictionary always to be used. Post cards: Yes.

All letters referring to this page which do not arrive at **140, Strand, London, W.C.**, by the *first post on Thursdays*, and are not addressed PRIZE EDITOR, will in future be disqualified and disregarded.

N.B.—Each paper must be signed by the author with his or her real name and address. A *nom de plume* may be added if the writer does not desire to be referred to by us by his real name. In the case of all prize-winners, however, the real name and address will be published.

### From Pharmacy to Farming.

(*Concluded from page* xxiv.)

It was seven when Joshua awoke, with a headache. He had slept two hours beyond his usual time of rising, and he sat on the edge of the bed, puzzled for a reason of his drowsiness. Hurrying over his toilet, he screwed down the plank, drew the carpet over it, and went out to the hay-fields. At eight he returned to breakfast.

"A queer go, Martha," he said to Mrs. Powell. "Blessed if I didn't oversleep myself this morn'. I can't be well; that's about the size on it. You know I gen'rally sleep sound's a top. Aint Edward down?"

"No; and he wasn't stirring, neither, when I got up. I'll go and call him."

In a moment she descended, with a look of perplexity upon her face.

"He's up and gone, and has took his bag!" she said.

"What!" cried Joshua, dropping his knife and fork, and changing colour. Then, with an unintelligible exclamation, he fled to his room, and seized the screwdriver. Quickly drawing the screws, he pulled up the board, and unlocked the safe. A cry brought Mrs. Powell to his side.

"Go away!" he shouted, slamming the door of the safe.

"I bin robbed! I bin robbed! Edward's broke into my room in the night—Above knows how!—and took several hundreds. Oh! what's to be done! What's to be done!"

"Are you sure?" asked Mrs. Powell, at the door.

"Do my eyes deceive me, wench? Out o' the way! I'm off arter him, straight away. Help me saddle the pony."

"But you don't know where he be gone yet. And hadn't you ought to tell the constable?"

"'Tis my money—my money as I wants," cried Joshua. "Ten to one he's gone up to London by the six express from Marley. I must be off there like thunder and lightning, to inquire o' the railway folk."

After a desperate gallop Joshua reached the station, and learned that a young man in a grey tweed suit had taken a ticket for town, and left by the six o'clock train. A train started in ten minutes. Directing a porter to take the panting pony to a stable, Joshua took a ticket. When he alighted at Paddington he was told to spend money upon a cab to take him to Edward's lodgings at Hackney. But there was no alternative if he wished to save time, for he was told that a journey by rail might be slower. He even went to the extremity of extravagance, and offered the cab-man an extra florin to drive quickly.

But, unfortunately, Edward was not at home, and his landlady said she had not seen him since he left her to go down into the country. In an agony of soul poor Joshua explained affairs to the cabman, and begged his counsel.

"If I was you, guv'nor, I'd go slick to Scotland Yard, and inform the p'lice," said the driver.

"Then drive me there," answered Joshua, wringing his hands, and re-entering the cab.

Meanwhile, Mrs. Powell had made an important discovery. She had found a bottle of queer-smelling stuff on the mantel-shelf in Edward's bed-room, and there was small doubt in her mind as to the nature of the drug. It was chloroform! Here then, she argued, was the clue to Joshua's sluggishness. His nephew had drugged and robbed him! Some hours later, when a detective arrived with Joshua, she thrilled with pride for her astuteness when the keen-eyed official concurred with her view.

"But," said he, "how did the young fellow get into the room? Was the door locked when you went to sleep, Mr. Harman?"

"That I can swear to," said Joshua. "'Tis possible that he got in by the window. A lightish chap might climb along the ivy; but it don't seem scarcely likely."

"Was your door locked on the inside when you got up this morning?" asked the detective.

"I can't be positive certain about that," answered Joshua, scratching his head. "I felt so tired and giddy-like, that, to tell 'e the truth, I took no notice. It strikes me, however, that if it had bin unlocked I should 'a noticed it."

The detective asked no more questions. He made some memoranda in a note-book, and took possession of the chloroform bottle before leaving the house.

It was an ill day for Joshua. He sat in the parlour till dark brooding over his misfortune, and refusing to eat tea or supper. It was the hardest blow that Fate had dealt him, and his head was bowed in abject misery. Eleven struck, but still he sat gazing vacantly at the floor, with his right arm hanging listlessly over the arm of his chair.

A few minutes before midnight he heard a sound in the room. Turning quickly, he confronted his nephew. The young man's face was deadly pallid in the moonlight, and he stood like one in a dream. With a low cry of surprise, Joshua rose, and turned the key in the lock.

"I'm not going to run away," said Edward, sitting down by the window.

"Where's my five hundred, you scoundrel?" cried Joshua, gripping his arm. "Give it back, and I'll say no more. I'll not send 'e to gaol so long's I get my money," he said.

"I know nothing about your money. I have read in the evening papers that you've been robbed, and I have come down at once lest folk should suspect me of taking it."

"Is it solemn truth, on your oath, that you're speakin'?" asked Joshua.

"Yes. I went away in a huff this morning. You were quick to suspect me, Uncle Josh."

"What about the bottle o' stuff in your room?"

"The chloroform? That is a preparation I use in cases of acute neuralgia in the face, to which I am subject at times."

"It's a queer job," said Joshua, looking keenly at his nephew. "How did you get out o' the house?"

"By the garden door, which I found unlocked."

"Unlocked! I can swear as I locked it last night."

"It was unlocked this morning."

"Then, if so be as you're speakin' the truth, we're no nearer findin' the thief than we was this morn'," groaned Joshua.

Edward sat silent for a moment.

"Did you sleep last night?" he asked his uncle.

"Arter twelve—not afore. I overslep' mysel' when I did get off, and I woke up feelin' mortal tired."

"Do you ever walk in your sleep?" said Edward eagerly, as light seemed to dawn on the mysterious affair.

"I did used to as a youngster," replied Joshua.

"Come with me down the garden," cried Edward, catching his arm, and leading him out.

"What's your notion?" asked Joshua in bewilderment.

Edward made no answer, but went straight to the lilac-bush near the gate, and closely examined the earth.

"Put your foot in that mark," he said. "Ah! it fits. And look here, someone's been digging!"

"Sure enough!" said Joshua.

Then Edward went for a spade, and turned over the earth.

"What's that?" he said. "Look for yourself. Is that your lost money?"

"Lor! if it ain't!" cried Joshua, kneeling down, and grabbing the notes with a childish chuckle.

"And now," said Edward, sternly, "the next time you lose some of your precious money don't be so quick to suspect an innocent man. You'll bank it, if you take my advice, and then, if a visitor sleeps in the house, you won't fret yourself half crazy thinking he's a thief. Good-night."

"Here, stop, Edward—stop, lad!" said Joshua, as the tears rolled down his furrowed cheeks. "Don't 'e go away like that, lad, and say as I've bin fooled by mysel'. I've bin onkind to 'e, and I owns it like a man. I—I'll give 'e good wages to come and help me w' the books. You be a sharp chap—a sharp chap! I'm gettin' dotty, Edward, and I wants a young fellow to look arter my interests. Don't go, Edward! I'll give 'e fifty, sixty pound a-year to act as bailiff. I'll soon learn 'e what to do. Come, lad, gi'e's your hand and say as it's a bargain!"

They went into the house together.

Geoffrey Mortimer.

## Figures and Tables

**Figure f1:**